# Research Progress in the Molecular Functions of Plant mTERF Proteins

**DOI:** 10.3390/cells10020205

**Published:** 2021-01-21

**Authors:** Pedro Robles, Víctor Quesada

**Affiliations:** Instituto de Bioingeniería, Universidad Miguel Hernández, Campus de Elche, 03202 Elche, Spain; probles@umh.es

**Keywords:** organellar gene expression, mitochondrial transcription termination factor, Arabidopsis, maize, chloroplast, mitochondria

## Abstract

Present-day chloroplast and mitochondrial genomes contain only a few dozen genes involved in ATP synthesis, photosynthesis, and gene expression. The proteins encoded by these genes are only a small fraction of the many hundreds of proteins that act in chloroplasts and mitochondria. Hence, the vast majority, including components of organellar gene expression (OGE) machineries, are encoded by nuclear genes, translated into the cytosol and imported to these organelles. Consequently, the expression of nuclear and organellar genomes has to be very precisely coordinated. Furthermore, OGE regulation is crucial to chloroplast and mitochondria biogenesis, and hence, to plant growth and development. Notwithstanding, the molecular mechanisms governing OGE are still poorly understood. Recent results have revealed the increasing importance of nuclear-encoded modular proteins capable of binding nucleic acids and regulating OGE. Mitochondrial transcription termination factor (mTERF) proteins are a good example of this category of OGE regulators. Plant mTERFs are located in chloroplasts and/or mitochondria, and have been characterized mainly from the isolation and analyses of *Arabidopsis* and maize mutants. These studies have revealed their fundamental roles in different plant development aspects and responses to abiotic stress. Fourteen mTERFs have been hitherto characterized in land plants, albeit to a different extent. These numbers are limited if we consider that 31 and 35 mTERFs have been, respectively, identified in maize and *Arabidopsis*. Notwithstanding, remarkable progress has been made in recent years to elucidate the molecular mechanisms by which mTERFs regulate OGE. Consequently, it has been experimentally demonstrated that plant mTERFs are required for the transcription termination of chloroplast genes (mTERF6 and mTERF8), transcriptional pausing and the stabilization of chloroplast transcripts (MDA1/mTERF5), intron splicing in chloroplasts (BSM/RUG2/mTERF4 and Zm-mTERF4) and mitochondria (mTERF15 and ZmSMK3) and very recently, also in the assembly of chloroplast ribosomes and translation (mTERF9). This review aims to provide a detailed update of current knowledge about the molecular functions of plant mTERF proteins. It principally focuses on new research that has made an outstanding contribution to unravel the molecular mechanisms by which plant mTERFs regulate the expression of chloroplast and mitochondrial genomes.

## 1. Introduction

Chloroplasts and mitochondria genetic systems are the relics of the genomes of ancestral prokaryotes, which were engulfed by a primitive eukaryotic cell to establish an endosymbiotic relationship [1,2]. As the immense majority of the genes of ancestral prokaryotic genomes were transferred to the nucleus, current genomes of chloroplast (plastome) and mitochondria (mitogenome) encode only a few dozen proteins, which are principally involved in ATP production through photosynthesis and oxidative phosphorylation, respectively. Moreover, the components of gene expression machineries, such as rRNAs, tRNAs, and ribosomal proteins, are also encoded by these organellar genomes and in the case of the plastome, harbor information for a bacterial-type multi-subunit RNA polymerase (plastid encoded polymerase, PEP). Nonetheless, all these proteins represent only a tiny fraction of the hundreds of proteins present in chloroplasts and mitochondria. Hence, the vast majority of them, including the components of organellar gene expression (OGE) machineries, are encoded by nuclear genes so they are translated in cytosolic ribosomes and imported into these organelles (reviewed in [3]). Consequently, OGE apparatuses are chimeric machines that result from hundreds of millions of years of evolution since endosymbiosis events took place [4,5]. The expression and regulation of organellar genes are crucial to chloroplast and mitochondria biogenesis, and hence, to plant development and growth. In line with this, the activity of these organelles must be very fine-tuned modulated to allow plants to adjust to various physiological demands and environmental cues. Notwithstanding, the molecular mechanisms governing OGE are still poorly understood.

It is noteworthy that the majority of the nuclear-encoded protein cofactors required for OGE are unrelated to either bacterial proteins or the proteins acting in the nucleus or the cytoplasm. Furthermore, they can bind DNA or RNA in vivo, which is consistent with their function in gene expression [6,7]. Along these lines, current OGE apparatuses exhibit a mixture of gene expression features from prokaryotes, eukaryotes, and new innovations [6]. This is especially relevant in chloroplast transcriptional machinery because chloroplasts utilize a functional, multi-subunit prokaryotic-type polymerase (PEP), having core subunits encoded by the organellar genome and accessory subunits encoded by the nuclear genome, including proteins with DNA/RNA binding domains. Besides this system with remnant prokaryotic components, plastids also harbor a single subunit, nuclear-encoded, bacteriophage-type polymerase (NEP). Plant mitochondria, in contrast, use only NEPs.

The research results of recent years reveal the important role that two of these types of accessory cofactors play in OGE: pentatricopeptide repeat (PPR) proteins and mitochondrial transcription termination factors (mTERF) [8,9]. PPRs and mTERFs are eukaryotic modular proteins that belong to the same family of helical-repeat proteins that are capable of binding nucleic acids [10]. They are principally targeted to mitochondria and chloroplasts. PPRs are characterized by the presence of degenerate helical repeats of around 35 amino acids and are associated with different OGE aspects (RNA transcription, splicing, editing, and translation) that develop after the endosymbiosis event [11]. Animal genomes usually harbor 10 genes that encode PPR proteins or fewer. In contrast, the PPR family has extended in plant genomes to become one of the largest families in land plants (e.g., 650 and 450 PPRs have been identified in rice and *Arabidopsis*, respectively) [12].

Like PPRs, mTERFs are also modular eukaryotic proteins characterized by a variable number of tandem repetitions of a 30 amino-acid motif named “mTERF” that comprises three α-helices domains [13]. The first mTERF protein to be characterized was human MTERF1, which specifically binds to a 28-bp region immediately downstream of the mitochondrial 16S rRNA gene [14]. MTERF1 received its name because it was initially thought to promote the transcription termination of heavy strands (HS) in human mitochondrial genes and it eventually gave its name to this family of proteins. Further studies show that human MTERF1 can also be implicated in both transcription initiation and the modulation of mitochondrial DNA replication [13]. However, later works do not support a function for MTERF1 in HS transcription termination. In vivo studies in an *Mterf1* knockout mouse model reveal that MTERF1 does not regulate mitochondrial rRNA synthesis at the HS. The binding of MTERF1 to the termination site would block the continuation of the light-strand promoter transcript, thereby preventing reading-through of this near full genome length transcript across its original promoter region to cause a minor effect on HS [15]. More recently, Shi et al. [16] demonstrated that MTERF1 acts as a DNA replication fork barrier to delay replication fork progression.

Additional animal MTERFs have also been characterized and grouped into four subfamilies in vertebrates, MTERF1–4, where all these proteins like MTERF1 are located in mitochondria [13,17]. Two of these subfamilies, MTERF3 and MTERF4, are also shared with invertebrates [18]. Like MTERF1, current knowledge does not support a role for MTERF2–4 in transcription termination, rather in other gene expression aspects, principally mitochondrial ribosome biogenesis in invertebrates and mammals [19,20,21]. Along these lines, MTERF2 binds to mitochondrial DNA (mtDNA) in a non-sequence-specific manner and coimmunoprecipitates with MTERF1 and MTERF3 [22,23]. MTERF2 and MTERF3, respectively, act as positive and negative regulators of mitochondrial transcription [23,24], whereas MTERF4 regulates the translation of mitochondrial genes by targeting rRNA 5-methylcytosine methyltransferase NSUN4 to the mammalian mitochondrial ribosome [19,20,25].

## 2. Plant *mterf* Defective Mutants Exhibit Developmental and/or Stress-Related Phenotypes

Unlike animals, plant mTERF functions are still poorly understood. Similarly to the aforementioned PPR proteins, land plant genomes harbor many more *mTERF* genes than animal genomes. In line with this, 35 mTERFs in *Arabidopsis* and *Capsicum annuum*, and 31 in maize, have been identified [26,27,28,29,30]. Plant mTERFs have been characterized to date principally from the isolation and analysis of *Arabidopsis* and maize mutants that show altered phenotypes. These mutant analyses have been instrumental for shedding light on the function of mTERF in plants, but also in animals (reviewed in [31]). Along these lines, plant *mterf* defective mutants usually exhibit stunted growth and paleness due to low chlorophyll levels when mTERFs are located in chloroplasts, and altered transcript abundance of chloroplast and/or mitochondrial genes. Furthermore, several of these mutants present developmental abnormalities. These phenotypes are likely the result of the perturbed biogenesis of chloroplasts [e.g., *Arabidopsis* mutants *bsm/rug2/mterf4* (*belaya smert/rugosa2/mterf4* [26,32]); *mda1/mterf5* (*mTERF* defective in *Arabidopsis1/mterf5* [28]); *mterf6* [33,34] and *mterf9/twr-2* [35]; and maize mutant *Zm-mterf4* [6]] or mitochondria (e.g., *Arabidopsis* mutants *shot1/mterf18* (*suppressor of hot1-4 1/mterf18* [36]) and *mterf22* [37]; and maize mutant *ZmSmk3* (*Zea mays small kernel 3* [38])). In line with all this, leaf morphology and internal anatomy are altered in mutants *bsm/rug2/mterf4*, *mda1/mterf5*, *mterf6*, and *mterf9*. Moreover, complete loss of *mTERF6* function results in seedling lethality [33], whereas the absence of SOLDAT10 (SINGLET OXYGEN-LINKED DEATH ACTIVATOR10 [39]) or BSM/RUG2/mTERF4 [26] function leads to embryo lethality in *Arabidopsis*. Knockout *Mterf3* or *Mterf4* mice are also embryo-lethal [19,24]. Altogether, these results underpin the essential functions of mTERFs in plant and animal development. It should be noted that the numbering and classification of animal and plant mTERFs is not based on the homology between metazoans and plant mTERFs. In line with this, the classification and numbering of *Arabidopsis* mTERFs is due to Tatjana Kleine [27], who sorted most mTERF proteins in this species into five groups on the basis of their hypothetical functions predicted using in silico co-expression analysis and gene ontology (GO) annotations. According to this, the first two clusters were named “chloroplast” (mTERF1–9) and “chloroplast-associated” (mTERF10–12) and these mTERF are principally involved in chloroplast gene expression, embryogenesis, and protein catabolism. The “mitochondria” group members (mTERF13–19) function in DNA and RNA metabolism in this organelle, whereas those of the “mitochondrion–associated” (mTERF20–22) are related to DNA and RNA metabolism in the mitochondria and the nucleus. Finally, the members of the group dubbed “others” (mTERF23–26), are expressed at low levels and may act in the nucleus. Despite the fact that metazoan and plant mTERFs are involved in RNA or DNA metabolism in the organelles, the levels of identity and similarity between plant and metazoan mTERF proteins are rather low [28,32,35].

It is worth mentioning that altered responses to different environmental stresses have also been reported for several *mterf* mutants, which supports an emerging role for plant mTERFs in abiotic stress tolerance (reviewed in [12,40]). Consistently with this, mutants *soldat10* and *shot1/mterf8*, respectively, display enhanced light sensitivity and tolerance to heat stress [36,39], whereas mutants *mda1/mterf5* and *mterf9/twr-2* are less sensitive to salt stress than the wild type (WT) [28,35], and mutants *mterf6* and *mterf10* are hypersensitive to salinity [41,42]. Numerous *Arabidopsis* mutants displaying enhanced or reduced tolerance to abiotic stresses also show altered sensitivity to the abscisic acid hormone (ABA). This is not unexpected given the fundamental role of ABA in plant adaptation to abiotic stress [43].

Taken together, the aforementioned mutant analyses reveal that mTERFs play a fundamental role in plant growth and development, and also in their response and adaptation to adverse environmental conditions (recently reviewed in [44]).

## 3. Deciphering the Molecular Functions of Plant mTERF

Linder et al. [17] described for the first time the presence of the mTERF family of proteins in photosynthetic organisms. Henceforth, 14 plant *mTERFs* have been characterized, albeit to a different extent, from the isolation and analysis of mutants in *Arabidopsis* (SOLDAT10/mTERF1 [39]; BSM/RUG2/mTERF4 [26,32]; MDA1/mTERF5 [7,28,45]; mTERF6 [33,34,41,46]; mTERF8 [47]; mTERF9 [35]; mTERF10, mTERF11 and mTERF12 [42]; mTERF15 [48]; SHOT1/mTERF18 [36] and mTERF22 [37]) and maize (Zm-mTERF4 and ZmSmk3 [6,38]). Notwithstanding, the first mTERF to be thoroughly studied in a photosynthetic organism did not belong to a land plant, but to the unicellular alga *Chlamydomonas reinhardtii* (mTERF-LIKE GENE OF CHLAMYDOMONAS1, MOC1 [49,50]). These numbers are still small considering that nearly 70 mTERFs have been identified in *Arabidopsis* and maize (see above).

**Figure 1:**
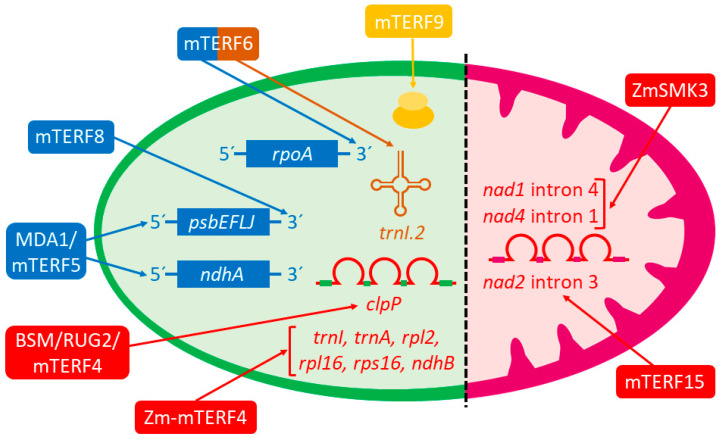
Schematic representation of the molecular functions of plant mTERFs. Colors of mTERF proteins and arrows refer to their activity as regulators of (i) transcription (blue), functioning as transcriptional pausing and stabilization (MDA1/mTERF5) or terminator (mTERF6 and mTERF8) factors of chloroplast genes (*psbEFLJ* polycistron, *ndhA* gene, and *rpoA* operon), (ii) group II intron splicing (red) of chloroplast (BSM/RUG2/mTERF4 and Zm-mTERF4) or mitochondria (mTERF15 and ZmSMK3) *clpP*, *trnI*, *trnA*, *rpl2*, *rpl16*, *rps16*, *nad1*, *ndhB*, *nad1*, *nad2* and *nad4* genes or (iii) translation (orange) of chloroplasts (mTERF9) promoting ribosomal assembly. mTERF6 also functions in *trnI.2* maturation (brown). Green and purple semi-ovals represent, respectively, chloroplasts and mitochondria.

Eight of the aforementioned mTERF proteins are targeted to chloroplasts (Zm-mTERF4, SOLDAT10/mTERF1, MDA1/mTERF5, mTERF8, mTERF9, mTERF10, mTERF11, mTERF12), five to mitochondria (MOC1, ZmSmk3, mTERF15, SHOT1/mTERF18, and mTERF22), and two possibly to both organelles (BSM/RUG2/mTERF4 and mTERF6). Nevertheless, a precise molecular function has been assigned to only a handful of them: BSM/RUG2/mTERF4, MDA1/mTERF5, mTERF6, mTERF8, mTERF9, mTERF15, Zm-mTERF4, and ZmSMK3 (Table 1 and Figure 1). The molecular mechanisms of action of the remaining mTERFs for which a mutant phenotype has been described remain to be discovered. The present results indicate that plant mTERFs participate in OGE regulation at transcriptional or post-transcriptional levels. In the former, the protein MDA1/mTERF5 promotes the transcription of chloroplast genes [7,45], whereas mTERF6 and mTERF8 are required for transcription termination in this organelle [33,46,47]. In the latter, BSM/RUG2/mTERF4, mTERF15, Zm-mTERF4, and ZmSmk3 participate in RNA splicing in chloroplasts or mitochondria [6,26,38,48], whereas mTERF9 promotes chloroplast ribosomal assembly and translation [51].

We report hereafter the molecular phenotypes of the *mterf* mutants described to date and the different experimental approaches carried out to elucidate the molecular function of mTERF proteins in photosynthetic organisms (for complementary reviews, see [44] and [52]).

## 4. *Arabidopsis* mTERF6 and mTERF8 Mediates the Transcription Termination of Chloroplast Genes

Chloroplast transcription is a complex process that involves two types of DNA-dependent RNA polymerases, as well as a number of accessory proteins encoded by the nucleus (see the Introduction; reviewed in [5]). As the transcription of plastome genes is fundamental for plant survival, it must be very carefully modulated. One of the least understood steps in regulating chloroplast RNA synthesis is transcription termination, a process that involves: (a) arrest of RNA synthesis; (b) release of the newly synthesized transcript; (c) separation of RNA polymerase from the DNA template [47]. Regulation of transcription termination is important to assure that RNA synthesis is not affected by the interference of transcripts from downstream genes and to, thus, avoid the formation of interfering antisense RNAs [53,54].

As previously mentioned, the founder member of the mTERF family was human MTERF1, to which the ability to terminate mitochondrial transcription in vitro was assigned [14]. Notwithstanding, the results obtained since then refute this idea. In fact, none of the remaining mammalian MTERFs function as termination factors of mitochondrial transcription (reviewed in [31]). In photosynthetic organisms, the detailed characterization of *Chlamydomonas* mTERF protein MOC1 revealed that it functions as a terminator of mitochondrial antisense transcription by binding to an octanucleotide sequence in mitochondrial rRNA coding module S3 [50].

In land plants, the first known example of a protein involved in the termination of plastid transcription was *Arabidopsis* RHON1, which terminates *rbcL* (encoding a large subunit of ribulose-1,5-bisphosphate carboxylase/oxygenase) transcription via a mechanism resembling that of the Rho factor of *Escherichia coli* [55]. Nonetheless, it was unknown whether, unlike animals, plant mTERFs would live up to their names by participating in transcription termination in chloroplasts and/or mitochondria. Recent elucidation of the molecular functions of *Arabidopsis* mTERF6 and mTERF8 has shown for the first time that mTERF proteins can terminate transcription in chloroplasts. The first piece of evidence came from the work by Romani et al. [33], who reported that recombinant mTERF6 can bind to a target dsDNA sequence located in the chloroplast isoleucine transfer (*trnI.2*) gene to promote in vitro transcription termination in that sequence (Table 1). Furthermore, coimmunoprecipitation experiments have revealed that mTERF6 also binds in vivo to its target sequence in the RNA of *trnI.2*. Interestingly, these authors discovered that *trnI.2* maturation was impaired in the *mterf6-1* albino mutant, which indicates that mTERF6 activity might also be fundamental for tRNA maturation (Table 1 and Figure 1).

Three years later, Zhang and colleagues demonstrated the mechanism by which mTERF6 terminates transcription in chloroplasts from the characterization of two additional *mterf6* mutants. These authors reported that mTERF6 directly associates in vitro and in vivo with a 3′-end sequence of the *rpoA* polycistron L23-L2-S19-L22-S3-L16-L14-S8-L36-S11-rpoA, which encodes some essential ribosomal proteins and the RpoA core subunit of PEP (Table 1 and Figure 1). These authors found that the transcript levels of PEP-dependent genes were down-regulated in the *mterf6-5* mutant compared to the WT, which suggests that PEP activity might be reduced in this mutant. Consistent with this hypothesis, the protein levels of RpoA were lowered in *mterf6-5* individuals, which prompted Zhang et al. [46] to investigate whether mTERF6 can terminate the expression of *rpoA* and other genes encoding the core subunits of PEP (*rpoB, rpoC1,* and *rpoC2*), as well as some other plastid genes. To this end, they designed primer sets to characterize the non-transcribed regions of the plastome to detect potential transcription read-through in the *mterf6-5* mutant. This was the case for genes *rpoA, petD, ycf5,* and *rbcL* because the transcripts from the non-transcribed spacer regions in these genes accumulated in *mterf6-5* at higher levels than in the WT. Afterward, they applied chloroplast chromatin immunoprecipitation (cpChIP) to investigate whether mTERF6 can bind to the 3′-end regions of genes *rpoA, petD, ycf5*, and *rbcL*. mTERF6 enrichment was reported at the 3′ ends of *rpoA, rbcL,* and *ycf5,* suggesting that the mTERF6 protein can bind in vivo to the 3′ termini of these genes. Nonetheless, affinity was higher for *rpoA*. By running an electrophoretic mobility shift assay (EMSA), they confirmed that mTERF6 binding to the 3′-end region of the *rpoA* gene was direct. Furthermore, a conserved sequence [ATT(N)_5_GT] in the 3′-end of genes *rpoA, rbcL,* and *ycf5* was identified, which suggests that mTERF6 can recognize specific sequences in its target genes. Interestingly, Romani et al. [33] had already reported that mTERF6 can specifically bind in vitro to the ATT(N)_5_GT sequence located within the *trnI.2* gene. The low expression in the *mterf6-5* mutant of *rpoA* polycistron and the *petD* gene, located downstream of the *rpoA* polycistron and transcribed in the opposite orientation to *rpoA*, prompted Zhang et al. [46] to hypothesize that mTERF6 may act as a roadblock or a bidirectional transcription-termination factor that would avoid transcription collision from two directions. Given that mTERF6 and the 3′-end regions of gene *rpoA* are well conserved among plant species, mTERF6 homologs may also function in transcription termination in these species [46].

mTERF8 is the second mTERF for which a role in transcription termination in chloroplasts has been demonstrated. In a paper published in 2020, Xiong and collaborators found that mTERF8, formerly pTAC15, a plastid transcriptionally active complex component [8], is localized in chloroplast nucleoids. Xiong et al. [47] showed that mTERF8 comigrates with RpoB, a core subunit of PEP, and specifically associates with the PEP complex. Analysis of the loss-of-function mutant *mterf8* revealed that impaired mTERF8 causes slightly decreased photosynthetic electron flow efficiency, likely as a consequence of a reduction in the accumulation of proteins PSII PsbA and PsbB. The levels of transcripts of several plastid genes in the *mterf8* mutant were altered, and the increased transcript levels of the *psbJ* gene were the most remarkable change compared to the WT. *psbJ,* as well as genes *psbE*, *psbF*, and *psbL* (respectively, encoding PsbJ, the α and β subunits of Cyt b_559_, and PsbL proteins, all of which are essential components of PSII) constitute an operon and, like *psbJ*, the other three genes were also up-regulated in *mterf8*.

When the expression of the *psbEFLJ* polycistron was analyzed by RNA blot employing a probe to span all the transcripts synthesized from this polycistron, a band of the same size was detected in the WT and *mterf8* plants. In contrast, a larger transcript was observed only in *mterf8*. These results suggest the existence of read-through transcription of the *psbJ* gene cluster and/or perturbed RNA processing in *mterf8*. By a similar experimental approach to that previously employed to investigate whether mTERF6 could terminate transcription (see above), Xiong and colleagues analyzed the potential transcription read-through in *mterf8*. Defective transcription termination in mutant *mterf8* was found as the transcription of the *psbJ* polycistron terminates downstream of the *psbJ* gene and farther than in the WT. Furthermore, circular RT-PCR and sequence analyses revealed that the termination site of the *psbJ* gene was located 95 nucleotides downstream of its stop codon in the WT, whereas its termination site was altered in *mterf8*. With EMSA and cpChIP analyses, Xiong et al. [47] discovered that mTERF8 specifically binds downstream of the stop codon of *psbJ* near the termination site. They also demonstrated in vitro that mTERF8 possesses transcription termination activity by acting specifically on the 3’ terminal region of the *psbJ* gene (Table 1 and Figure 1). As this region can form a stem-loop structure that resembles the terminators of Rho-independent transcription termination in *E. coli*, these authors proposed that mTERF8 could apply a similar mechanism to terminate *psbJ* transcription.

As mTERF6, mTERF8, and RHON1, respectively, terminate the transcription of genes *rpoA, psbJ,* and *rbcL* only, but not of other PEP-transcribed genes [46,47,55], these proteins seems to act as specific, rather than general, transcription termination factors.

## 5. *Arabidopsis* MDA1/mTERF5: A Protein That Plays a Dual Role in Transcriptional Pausing and the Stabilization of Chloroplast Transcripts

In addition to transcription initiation and termination, recent evidence shows that RNA elongation is also a widespread regulated process in bacteria and metazoan transcription [56]. Indeed, the extension of the nascent transcript is not a continuous process and the RNA polymerase usually pauses at some specific DNA sequences. This pausing delays transcript elongation and performs important regulatory functions in gene expression (e.g., facilitating the integration of cellular signals into genes by acting in signal-responsive pathways [56]). Nevertheless, it was unknown whether transcriptional pausing also occurred in chloroplasts and, if so, whether it would have a regulatory function. This scenario has recently changed due to the comprehensive work performed by Ding et al. [45], who recently elucidated the molecular mechanisms of *Arabidopsis* MDA1/mTERF5 action.

These authors characterized T-DNA knockout *mterf5* mutants of the *MDA1/mTERF5* gene, which displayed reduced growth, pale green leaves, and a defective PSII function. In line with this, *mterf5* individuals showed a severe reduction in the levels of the core subunits of PSII, as well as other key photosynthetic complexes, compared to the WT. Their study of the expression of plastid genes revealed that the transcript levels of *psbEFLJ* polycistron (see above) were marked lower in the *mterf5-1* mutant, whereas no significant differences were reported for the other studied genes. Furthermore, of all the investigated genes, only the expression patterns of the transcripts produced by the *psbEFLJ* promoters, as well as the transcriptional initiation rate for *psbEFLJ,* were perturbed in the *mterf5* mutants. All this suggests that MDA1/mTERF5 specifically regulates *psbEFLJ* transcription (Figure 1).

By means of cpChIP-sequencing using *35S:mTERF5-HA* transgenic plants and EMSA, by employing a recombinant MDA1/mTERF5 protein, it was observed that MDA1/mTERF5 binds directly to a region upstream of the *psbE* initiation codon. DNase I footprinting and EMSA analyses identified a 21-bp sequence as the MDA1/mTERF5 target sequence; it spanned from +30 to +51 in relation to the transcription start site (TSS) of *psbEFLJ* for P1 (Table 1). Interestingly, MDA1/mTERF5 specifically binds to dsDNA but not single-stranded DNA of the +30 to +51 target sequence.

To investigate whether MDA1/mTERF5 causes transcriptional pausing around the promoter region of *psbEFLJ*, Ding and colleagues used an in vitro transcription system, which was developed to monitor transcriptional activities from initiation to RNA extension. They concluded that MDA1/mTERF5 causes transcriptional pausing around +40 in relation to the TSS of *psbEFLJ* (Table 1). Furthermore, a global run-on sequencing (GRO-seq) data analysis confirmed that transcriptional pausing occurs at the promoter region of *psbEFLJ* in vivo, and this transcriptional pausing is dependent on the binding of MDA1/mTERF5 to its target sequence. Remarkably, the re-analysis of the public GRO-seq data revealed that transcriptional pausing occurs at nearly 30% of *Arabidopsis* plastid genes, which indicates that this is a general feature of plastome genes.

Using cpChIP with antibodies to recognize RpoB, the β subunit of PEP, they also detected, in the WT, a high occupancy of the PEP complex near the *psbEFLJ* promoter, but not in other regions of this polycistron or in *mterf5-1*. The use of different state-of-art molecular methods has revealed that (a) pTAC6 is an interaction partner of MDA1/mTERF5; (b) most pTAC6 is associated with the PEP complex, but also exists outside this complex; (c) pTAC6 is not required for MDA1/mTERF5 binding to its target sequence in *psbEFLJ* and hence, (d) pTAC6 would not be involved in transcriptional pausing. Nonetheless, MDA1/mTERF5 recruits pTAC6, but not the PEP complex, to the transcriptional pause region of *psbEFLJ.* Interestingly, Ding and colleagues discovered that after transcription initiation, the amount of pTAC6 associated with RpoB in the PEP complex increased, whereas the pTAC6 level associated with MDA1/mTERF5 lowered. This suggests that pTAC6 recruited by MDA1/mTERF5 is assembled in the PEP complex. These authors proposed that the additional recruiting of pTAC6 into the PEP complex would enhance PEP activity by facilitating transcription elongation and accelerating transcription. Hence, MDA1/mTERF5 would function as a positive regulator of *psbEFLJ* transcription.

Bacteria transcriptional regulators or pausing factors bind to non-template DNA or hairpin RNA. However, MDA1/mTERF5 binds to the dsDNA target sequence, which suggests that MDA1/mTERF5 transcriptional pausing likely represents a different regulatory mechanism [45]. Therefore, this may be a chloroplast invention that developed through evolution for accurate and specific *psbEFLJ* expression regulation, which is fundamental for PSII functioning.

Shortly after the publication of the aforementioned work, Méteignier et al. [7] reported novel aspects about the function of MDA1/mTERF5 by means of a reverse genetics approach combined with several molecular and biochemical techniques. They characterized an *Arabidopsis* T-DNA knockout line, dubbed *mda1-2* (also named *mterf5-1* by Ding et al. [45]; see above), which was previously described by Robles et al. [28] (see Section 2), and a second mutant allele of *MDA1/mTERF5*, *hcf111-1* [57]. *mda1-2* plants showed a moderate loss of the PsaD subunit of PSI and a substantial reduction in the subunits of complexes PSII and NDH compared to the WT. Consistent with this, photosynthetic capacities of mutants *mda1* and *hcf111-1* decreased. Moreover, the expression analysis of chloroplast genes in the *mda1-2* and *hcf111-1* plants revealed that the genes of the *psbEFLJ* polycistron, as well as genes *ndhA* and *ndhI* from the *ndhH* cluster, were specifically affected (they were all down-regulated). Like Ding et al. [45], Méteignier and collaborators studied the pattern and abundance of the transcripts of *psbEFLJ* by RNA blotting, and also that of the *ndhH* gene cluster. They mapped their 5´and 3´ends by cRT-PCR. As regards the *psbEFLJ* polycistron, in *mda1-2* plants, these authors observed decreases in the levels of two transcripts of 1.1 and 1.4 kb compared to the WT, which was more marked for the former. Furthermore, the 1.1 kb transcript was found to be a 5′-end processed *psbEFLJ* mRNA, and its mapping termini revealed that the frequency of the principal *psbE* mRNA termini had diminished in *mda1-2* compared to the WT. This was more pronounced for the 5′- than for the 3′-end. Afterward, a reduction in the abundance of the different mRNAs containing genes *ndhA* and *ndhI* was also observed in *mda1-2*. Interestingly, the less abundant transcripts in *mda1-2* started with an *ndhA* 5′-end and its mapping termini showed that the frequency of the predominant *ndhA* 5′-end is reduced in *mda1-2.* Therefore, the authors concluded that MDA1/mTERF5 is required principally for the stability of the 5′-end of mature *psbEFLJ* and *ndhA* mRNAs by promoting the in vivo accumulation of the transcripts containing processed *psbE* and *ndhA* 5′-ends (Table 1). To determine if changes in transcription could contribute to the lower steady-state levels of transcripts *psbE* and *ndhA*, Méteignier and collaborators quantified *psbE* and *ndhA* transcription activity and transcript abundance in *mda1-2* and the WT. Compared to the WT, they observed in *mda1* that the *psbE* and *ndhA* transcription rates had lowered, although the reduction in the mutant of the steady-state levels of the *psbE* and *ndhA* transcripts was much more marked. Therefore, these results indicate that MDA1/mTERF5 promotes *psbE* and *ndhA* transcription, but principally contributes to the posttranscriptional stabilization of their 5′-end processed mRNAs in vivo (Table 1 and Figure 1).

Coimmunoprecipitation experiments and the identification of proteins by LC-MS/MS revealed that MDA1/mTERF5 is found in high molecular weight complexes and associates with TAC components, including the core subunits of PEP. Méteignier and colleagues investigated the capacity of MDA1/mTERF5 to bind nucleic acids in vitro and in vivo by gel mobility shift assays, RNA immunoprecipitation sequencing (RIP-seq), and DNA immunoprecipitation (DIP)-qPCR experiments on solubilized chloroplasts, and they drew several conclusions. First, the MDA1/mTERF5 protein is able to bind dsDNA, and also ssRNA to a lesser extent. Second, MDA1/mTERF5 is not the RNA binding protein that stabilizes the termini of mature *psbEFLJ* and *ndhA* processed mRNAs, but promotes the in vivo binding of an RNA binding protein, likely a PPR protein, to the 5′-end of processed *psbE* and *ndhA* mRNAs. Third, MDA1/mTERF5 binds specifically to DNA regions in genes *psbE* and *ndhA*. Furthermore, they precisely mapped the binding site of MDA1/mTERF5 in genes *psbE* and *ndhA.* They found 27-nt DNA sequences located near the promoters of both genes. These target sequences were located −96/−70 from *psbE* ATG (overlapping the transcriptional pausing site identified by Ding et al.) and +110/+136 within the *ndhA* ORF, and the affinity for the *ndhA* binding site was weaker than for *psbE*.

By considering the aforementioned results and those by Ding et al., Méteignier and colleagues proposed a model for the MDA1/mTERF5 function in the control of *psbE* expression. According to this, MDA1/mTERF5 binding to DNA near the promoter region of *psbE,* and downstream of a potential PPR binding site, would prevent the formation of RNA secondary structures that are deleterious for the potential PPR binding to the 5′ end of *psbE* mRNA. This would post-transcriptionally stabilize the processed *psbE* mRNAs. *ndhA* expression might be regulated by a similar mechanism. Therefore, MDA1/mTERF5 performs a dual function in gene expression regulation: it stimulates *psbE* and *ndhA* transcription, and also stabilizes their post-transcriptionally processed mRNAs.

## 6. mTERF4 Functions in Group II Intron Splicing in *Arabidopsis* and Maize Chloroplast Genes

Some tRNA and protein encoding genes of chloroplasts and plant mitochondria harbor group I and II introns; the latter, in turn, are divided into a and b subclasses depending on their primary and secondary structures, and their different splicing mechanisms. These introns are processed and spliced by the combined action of a set of nucleus-encoded RNA-binding proteins, including, among others, maturases, CRM (chloroplast RNA splicing and ribosome maturation) domain-containing proteins, and PPRs [58]. Several mTERFs have also been recently found to participate in organellar intron splicing.

*Arabidopsis* mTERF4 was the first plant mTERF reported to function in plastid splicing. Babiychuk and colleagues [26] analyzed the subcellular localization of the 35 members of the mTERF family in *Arabidopsis* using GFP fusions to find that 11 were chloroplast-targeted and that T-DNA insertions in four of these caused embryo lethality. The insertional lethal alleles of one of these genes, *mTERF4*, led to arrested embryo development and produced immature white seeds, which was why the mutant was named *belaya smert* (*bsm*, white dead in Russian). Viable mutant alleles of *mTERF4*, named *rugosa2*, have also been identified and characterized, their most conspicuous phenotypic trait being the presence of green and yellowish leaf sectors in their vegetative leaves, probably as a result of perturbed chloroplast biogenesis [32]. *bsm* stable shoot cultures, which grow very slowly, can be maintained in vitro by the exogenous supplementation of phytohormones in order to perform further studies. In *bsm* albino shoots, the transcripts of the PEP-dependent *rrn16S*, *rrn23S*, *rbcL*, and *atpA* chloroplast genes were undetectable. In contrast, the transcript levels of the *clpP* gene increased in *bsm* cells compared to the WT, while the second *clpP* group IIa intron was not spliced in the mutant. Like *clpP*, the group IIa introns present in the *atpF*, *rpl2*, and *rps2* genes were not spliced in *bsm*. The authors proposed a direct role of BSM in the splicing of the second *clpP* group IIa intron (Table 1 and Figure 1), which is thought to be plastid MatK maturase-independent because the inhibition of plastid translation with spectinomycin abolishes the splicing of *atpF*, *rpl2*, and *rps12* group IIa introns, but not that of the *clpP* gene second intron.

Shortly after the work of Babiychuk and colleagues, a role for mTERF4 in organellar splicing was reported in a second plant species. Hammani and Barkan [6] worked with maize and found two insertional non-photosynthetic mutants of the Zm-*mTERF4* gene, the ortholog of the *BSM/RUG2/mTERF4 Arabidopsis* gene. Immunoblotting assays revealed that Zm-mTERF4, like BSM, is chloroplast-localized, primarily in the stromal fraction. Northern blotting demonstrated that plastid 16S and 23S rRNAs levels lowered in the Zm-*mterf4* mutants compared to the WT, which probably causes strong plastid ribosome deficiencies, as widely reported in other albino maize mutants [59,60,61,62]. Using immunoprecipitation with a Zm-mTERF4 antibody against several stromal extracts, followed by RIP-chip analyses, Hammani and Barkan identified RNA group II introns sequences from the transcripts belonging to 16 different plastid genes as ligands of Zm-mTERF4. In order to know if splicing was compromised in these transcripts, the authors used RNA blotting hybridizations with exon probes and qRT-PCR to compare the ratio of the spliced and unspliced introns in the Zm-*mterf4* mutants with those of the WT, pale green *hcf7* [63], and albino *iojap* [64] maize mutants. These mutants were included because they displayed defective plastid ribosomes, which brought about pleiotropic effects on the splicing of plastid introns [65]. Altogether, these results led the authors to classify introns into three categories: (i) *trnI*, *trnA*, *rpl2*, *rpl16*, *rps16*, and *ndhB* introns that coimmunoprecipitate with Zm-mTERF4, whose splicing is strongly increased by Zm-mTERF4; (ii) *ndhA*, *rps12*, *rpl16*, *rps16*, and *ndhB* introns that are weakly enriched in coimmunoprecipitation with Zm-mTERF4, whose splicing requires no Zm-mTERF4 activity; (iii) *trnK*, *trnG*, *trnV*, *ycf3-1*, *petB*, and *petD* introns, highly enriched in Zm-mTERF4 coimmunoprecipitation, but their splicing defects are slight or difficult to evaluate due to secondary effects caused by loss of plastid ribosomes.

By using sucrose gradient-fractionated chloroplast stroma, Hammani and Barkan found Zm-mTERF4 in particles within the same size range as large intron-containing complexes, where group II intron splicing factors have been identified. To confirm that Zm-mTERF4 is found in these particles containing splicing factors, Zm-mTERF4 was immunoprecipitated from chloroplast stroma, and both pellet and supernatant fractions were analyzed by immunoblotting with antibodies against several chloroplast splicing factors. The chloroplast RNA binding proteins that shared intron ligands with Zm-mTERF4 (CAF2, CFM2, CFM3, CRS1, WHY1, RNC1, THA8, and WTF1) coimmunoprecipitated with Zm-mTERF4. However, other proteins like PPR10 and CRP1, which bind non-intronic regions on plastid transcripts, were not detected in immunoblots.

Taken together, the results by Hammani and Barkan convincingly demonstrate that Zm-mTERF4 participates in group II intron splicing in chloroplasts (Table 1 and Figure 1), and show that mTERF4 function has been conserved in dicots (*Arabidopsis*) and monocot (maize) plants.

## 7. *Arabidopsis* mTERF15 and Maize ZmSmk3 Are Required for Intron Splicing in Mitochondria

Nine mitochondrial genes of *Arabidopsis* require intron splicing for the complete maturation of their transcripts, which comprises 23 intron-splicing events. Genes *rps3*, *cox2*, *ccmFc*, and *rpl2* contain a single intron, while genes *nad1*, *nad2*, *nad4*, *nad5*, and *nad7*, which are required for normal mitochondrial complex I function, contain multiple introns [66].

*Arabidopsis* mutant *mterf15*, defective in the plant-specific and mitochondrial-localized mTERF15 protein, displays stunted growth and development, small organs, delayed flowering, and sterility. These phenotypic traits may result from defective mitochondrial development and/or activity because the mTERF15 function is required for normal mitochondria biogenesis and membrane integrity [48]. To gain insight into the molecular function of mTERF15, Hsu and colleagues performed in vitro binding studies with dsDNA-cellulose and Northwestern blot analyses to investigate the dsDNA- and RNA-binding abilities of the mTERF15 protein. They found that mTERF15 functions as an RNA-, but not as a DNA-, binding protein. Using a set of primers designed by de Longevialle et al. [67], they examined by qRT-PCR the 23 mitochondrial splicing events in the *mterf15* mutant, WT, and transgenic plants that complement the mutant phenotype. The only altered RNA splicing event in the *mterf15* mutant was that of intron 3 of *nad2,* which was significantly reduced, as later confirmed by Northern blot. These authors also detected the accumulation of un-spliced *nad2b* transcripts in *mterf15* plants, which is consistent with defective intron 3 splicing because mature *nad2* mRNA contains five exons from two different transcripts, *nad2a* (exons 1 to 2 and intron 1) and *nad2b* (exons 3 to 5 and introns 3 and 4), as formed through one trans-splicing and three cis-splicing events. The interaction of mTERF15 with *nad2* intron 3 was confirmed by Northwestern blotting and its RNA-binding capacity by RNA-EMSA, specifically with a fragment spanning part of the sequence of the domain I intron (Table 1 and Figure 1). As *nad2* encodes a subunit of mitochondrial complex I of the electron transport chain, Hu and colleagues investigated complex I formation and activity in the *mterf15* mutant. They found that a *nad2* intron 3 splicing defect disrupts complex I formation and decreases its activity.

More recently, a recessive mutant of maize has been characterized, *ZmSmk3*, which harbors a *Mutator* transposon inserted in the promoter of the orthologous gene of *Arabidopsis mTERF15* [38]. ZmSMK3 is required for kernel development (embryo and endosperm development are arrested in the *Zmsmk3* mutant) and seedling growth because those germinated seedlings grow very slowly. With a GFP construct, Pan and colleagues found that ZmSMK3 is targeted to mitochondria. Their investigation by RT-PCR of the potential changes in the levels of 35 mitochondrial gene transcripts in the mutant and the WT revealed a drastic reduction in the levels of the *nad1* and *nad4* mature transcripts in *Zmsmk3*. On the contrary, the RNA precursors of the *nad1* and *nad4* mature transcripts had substantially increased. By qRT-PCR, the authors evaluated the splicing efficiencies of 22 mitochondrial introns to find that it was drastically reduced only in *nad1* intron 4 and *nad4* intron 1 in *Zmsmk3* compared to the WT (Table 1 and Figure 1). NAD1 and NAD4 are core components of mitochondrial complex I and, similar to the *Arabidopsis mterf15* mutant (see above), *Zmsmk3* plants also exhibit deficient mitochondrial complex I assembly and activity, as well as impaired mitochondrial function.

Pan and colleagues propose that ZmSMK3 may function in the recognition of precursor *nad4* and *nad1* mRNA, and in the maintenance of the *nad4* and *nad1* conformation for intron splicing with the cooperation of other factors. Furthermore, these authors noticed that despite the functional conservation of mTERF15 and ZmSMK3, and the high similarity of their target introns (*nad1* intron4, *nad2* intron3, and *nad4* intron1) in *Arabidopsis* and maize, these two proteins acted on different introns in these species. This finding could be attributed to differences in the structure of proteins because mTERF15 has five mTERF motifs, whereas ZmSMK3 only has two. Hence, divergence in target intron splicing would result from the variation in their protein structure.

## 8. *Arabidopsis* mTERF9 Protein Promotes Chloroplast Ribosomal Assembly and Translation

The *Arabidopsis mterf9* mutants show defective vegetative development and altered responses to salt [35] and photo-oxidative stresses [68]. The mTERF9 protein, also known as TWIRT1 [69], is localized to chloroplasts [26,68]. In a very recent work, published this year, Méteignier and collaborators, who already contributed to elucidating the molecular mechanisms of MDA1/mTERF5 function [7], have performed a comprehensive analysis of the molecular function mTERF9 [51]. In this work, these authors find that the loss of function of mTERF9 causes a pleiotropic photosynthetic deficiency by comparing the PS I and PSII activity of the *Arabidopsis mterf9* mutant and the WT. The use of an mTERF9-GFP fusion construct confirmed that the mTERF9 protein is targeted to chloroplasts and its emission specifically co-localized with the RAP-RFP nucleoid marker. Immunoblotting assays show that levels of most of the chloroplast protein complexes tested are reduced more than 50% in the *mterf9* mutant compared to WT and complemented *mterf9* plants (CP, with a WT copy of the mTERF9 gene). Furthermore, the de novo synthesis of the chloroplast proteins RbcL and D1 is also decreased in this mutant, indicating that accumulation and translation of chloroplast proteins are impaired in *mterf9*. Using qRT-PCR, the authors find that the steady-state levels of chloroplast gene transcripts or their splicing are not affected in *mterf9* plants, with the exception of 16S and 23S rRNAs, constituents of the small and large plastid ribosomal subunits, respectively, whose levels were significantly reduced compared with the WT. To investigate a possible defect in ribosome assembly, as suggested by defective accumulation of rRNAs, Méteignier et al. performed sucrose gradient fractionation of stroma from *mterf9*, WT, and CP plants and polysome analysis from sucrose gradients. The results show that mTERF9 associates with chloroplast ribosomes and, in turn, promotes mRNA association with them, indicating that this protein functions in translation. Furthermore, RIP assays reveal that mTERF9 binds the 16S rRNA in vivo and to a lesser extent, the 23S rRNA. Next, the authors identified, through coimmunoprecipitation of WT and CP stromal extracts treated or not with RNase, the mTERF9 protein interactome, made up of more than 150 proteins and highly enriched with proteins involved in chloroplast ribosome biogenesis. Unexpectedly, chaperonins from the CPN60 family are some of the mTERF9 in vivo protein interactants, and Méteignier and collaborators demonstrated, by using a yeast two-hybrid assay, that mTERF9 and CPN60 chaperonins physically interact. Altogether, these results prompted these authors to propose that mTERF9 would recruit the CPN60 chaperonin complex to chloroplast ribosomes during translation to help folding of nascent proteins. Nonetheless, they do exclude that, alternatively, mTERF9 might be itself a substrate of the CPN60 complex.

This recent work extends the known repertoire of plant mTERF functions to translation, beyond their roles in the control of OGE at the RNA level, and similar to metazoan mTERFs, since MTERF3 and MTER4 are involved in mitochondrial ribosomal assembly and therefore, translation [19,20,21,25].

## 9. Conclusions

The gene functions that persist in the chloroplast and mitochondria genomes are essential for the activity of these organelles and, consequently, for plant life. Therefore, OGE regulation must be carried out very precisely. Given the marked genomic erosion suffered by the genomes of the endosymbionts from which chloroplasts and mitochondria originate, OGE control is principally performed by proteins encoded by nuclear genes. Of these, nucleic-acid binding proteins with a modular structure, such as PPRs and mTERFs, play a fundamental role. The molecular functions of PPR in plant OGE control, especially in RNA editing, have been extensively studied for quite some time [70,71]. However, those of plant mTERFs have only recently started to emerge. We have reviewed herein the works that mainly contributed to shed light on plant mTERF molecular functions. Their results show that mTERFs operate at different levels in regulating OGE: (i) stimulating transcription and stabilizing post-transcriptionally processed mRNA (MDA1/mTERF5); (ii) terminating transcription (mTERF6 and mTERF8); (iii) promoting tRNA maturation (mTERF6); (iv) intron splicing (BSM/RUG2/mTERF4, Zm-mTERF4, mTERF15, and ZmSMK3; or (v) chloroplast ribosomal assembly and translation (mTERF9). As mTERFs hitherto molecularly characterized in detail are only a small fraction of those identified in model plants, such as *Arabidopsis* and maize, in forthcoming years, new works are expected to expand our knowledge about the biochemical and molecular functions in which plant mTERF participate.

## Figures and Tables

**Table 1:** Plant mTERFs molecularly characterized.

mTERF	Species	Localization	Molecular Function	Target ^1^	Reference
BSM/RUG2/mTERF4	*Arabidopsis thaliana*	C, M	Splicing of the second *clpP* group IIa intron	Binds non-specifically to chloroplast DNA	[26,32]
MDA1/mTERF5	*Arabidopsis thaliana*	C	Transcriptional pausing in the *psbEFLJ* chloroplast polycistron	A sequence spanning from +30 to +51 in relation to the transcription start site of *psbEFLJ* operon for promoter 1	[45]
			Stabilization of the 5′-end of mature *psbEFLJ* and *ndhA* mRNAs	DNA sequences located near the *psbE* and *ndhA* promoters	[7]
mTERF6	*Arabidopsis thaliana*	C	Maturation and promotion of transcription termination of chloroplast isoleucine transfer RNA (*trnI.2*)	*trnI.2* dsDNA sequences; *trnI.2* RNA	[33]
			*rpoA* polycistron transcription termination factor	3′-end sequence of the *rpoA* polycistron	[46]
mTERF8	*Arabidopsis thaliana*	C	Transcription termination of the chloroplast *psbEFLJ* polycistron	Downstream of the stop codon of *psbJ* gene near the termination site	[47]
mTERF9	*Arabidopsis thaliana*	C	Promotion of chloroplast ribosome assembly and translation	16S, and to a lesser extent, 23S rRNAs	[51]
mTERF15	*Arabidopsis thaliana*	M	Mitochondrial *nad2* intron 3 splicing	Domain I of mitochondrial *nad2* intron 3 transcript	[48]
Zm-mTERF4	*Zea mays*	C	Splicing of chloroplast group II introns	Chloroplast group II introns	[6]
ZmSMK3	*Zea mays*	M	Mitochondrial *nad1* intron 4 and *nad4* intron 1 splicing	Not determined	[38]

^1^ Experimentally demonstrated by protein-nucleic acids binding assays. C: chloroplast; M: mitochondria.

## Abbreviations

OGE—organellar gene expression; PPR—pentatricopeptide repeat proteins; mTERF—mitochondrial transcription termination factor; SOLDAT10—SINGLET OXYGEN-LINKED DEATH ACTIVATOR10; BSM/RUG2—BELAYA SMERT/RUGOSA2; MDA1—mTERF DEFECTIVE IN ARABIDOPSIS 1; SHOT1—SUPPRESSOR OF HOT1-4 1; MOC1—mTERF-LIKE GENE OF CHLAMYDOMONAS1.
